# Congenital Non-Secretion of Urine

**Published:** 1857-08

**Authors:** Thomas Hall

**Affiliations:** Toulon, Ill.


ARTICLE II.—*Congenital Non-Secretion of Urine.* By THOMAS HALL, M. D., Toulon, Ill.

December 1, 1856, 9 o'clock P. M.—Called in consultation to see a child five days old, and had not passed any urine. On examination, a large tumor is felt filling the pelvis and extending half an inch above the umbilicus, tolerably hard and slightly elastic; the integuments dark red, and have somewhat of the appearance that subjacent inflammation usually imparts. A probe was introduced into the bladder without any difficulty, but no urine followed its withdrawal. A grooved directer was next introduced, under the impression that urine might pass down the channel, but none escaped. The child was next put in a warm bath for about five minutes; no diminution of the swelling, nor softening followed its use. A common sized female catheter was next introduced—the child seemed to suffer but little more from its use than from the probe—and not a drop of blood exuded during these operations; no fluid, however escaped, and the catheter was withdrawn, under the impression we had that something had got into it. We found our opinion verified, for on blowing into the catheter something like half melted lard escaped from every hole. We now expressed an opinion that the child could be relieved, as the catheter had been loaned, and our conclusion was that melted lard, in the absence of oil, had been used to lubricate it. After washing the catheter it was again introduced, but no urine escaped. The child now struggled somewhat violently, and some viscid mucus began to appear at the end of the catheter. With the assistance of a probe, about a teaspoonful was removed. When no more could be extracted, the catheter was withdrawn, and again an exudation of lard-like matter took place on our blowing into the catheter.

The general appearance of the child was that of perfect health—it nursed well, the bowels were regular, and the discharges natural; no appearance of drowsiness or distress.

Dec. 2—9 o'clock A. M. No apparent change since last night, except the integuments over the tumor are somewhat darker colored. On introducing the catheter, a few drops of urine, or what was judged to be urine, escaped. A syringe, which we had previously nicely adjusted to the catheter, was now used, so as to form a vacuum, hoping by this means to remove some of the accumulated mucus. We succeeded in filling the catheter, but having to withdraw it every time and blow it out, the advantage, to our minds, was not sufficient to compensate for the suffering occasioned by repeated catheterism. Warm fomentations and poultices, which had been ordered last night, were continued. Three minims of spt. ætheris nit. in a little water were ordered every three hours. The child still appears in perfect health, nurses well, and all the organs, except the kidneys and bladder, performing their functions properly.

Five P. M. The diaper moistened about the size of a dollar once since morning. Continued the treatment. The child's brows are occasionally knit, otherwise appears well, and nurses freely, the bowels moving frequently and are of a watery character, but no urinary odor can be detected.

Dec. 3—8 o'clock A. M. The infant evidently approaching its end, breathing rapidly and with considerable difficulty. The abdomen generally much enlarged since last night, and the integuments assuming a gangrenous appearance; the superficial veins visible over the whole of the lower half of the abdomen. Nothing ordered.

Died at 4 P. M.

Post-mortem at 9 A. M., 4th of December. The integuments covering the lower half of the abdomen of a very dark color; the superficial veins much enlarged and distinctly visible; the tumor much softened and apparently reduced in size. On opening the abdomen a large quantity of serum escaped; no marks of inflammatory action on the bowels, but the small intestines were united by firm bands to the parieties of the abdomen. The peritoneum covering the bladder, and extending into the right and left iliac regions, highly inflamed. Both ureters were pervious, and pus and mucus exuded on pressure. On dissecting the bladder, we found its coats thickened and the inflammation extended through all its tissues, except the mucous lining, which was completely destroyed. The bladder contained about one ounce of pus. The uterus was inflamed, and contained pus and mucus. The kidneys, which weighed six drachms and ten grains, and six drachms and eighteen grains, respectively, were inflamed through the greater part of their substance, and contained some pus and serum, but no traces of urine, at least as far as we were able to determine.

There was no time for any further investigations, the funeral taking place at 10 o'clock.

